# Bladder Cancer–Specific Nuclear Matrix Proteins-4 May Be a Potential Biomarker for Non-Muscle-Invasive Bladder Cancer Detection

**DOI:** 10.1155/2018/5609395

**Published:** 2018-09-10

**Authors:** Zhi-Yong Wang, Hong-Yang Li, Hao Wang, Qiang Chi, Ying Liu, Xiu-Ming Li

**Affiliations:** ^1^Urology Department, Affiliated Hospital of Chengde Medical University, Chengde, 067000 Hebei, China; ^2^Urology Department, Cangzhou People's Hospital, Cangzhou, 061001 Hebei, China

## Abstract

**Aims:**

Bladder cancer–specific nuclear matrix protein-4 (BLCA-4) is a protein expressed mainly in bladder cancer tissues. Therefore, the aim of this study was to investigate its assisting diagnostic potential in non-muscle-invasive bladder cancer (NMIBC).

**Methods:**

Twenty patients with NMIBC, 20 with benign prostatic hyperplasia (BPH), and 20 normal controls were included in this study. Blood and urine samples were collected from all patients. Moreover, cancer foci and adjacent tissue samples were collected from NMIBC patients, and normal bladder tissue samples were collected from patients with BPH. A competitive enzyme-linked immunosorbent assay was used to determine the BLCA-4 level in serum and urine, and immunohistochemistry was used to examine BLCA-4 expression in bladder cancer, adjacent, and normal tissues.

**Results:**

Median urinary BLCA-4 levels in the NMIBC, BPH, and normal control groups were 0.759 ng/mL, 0.309 ng/mL, and 0.171 ng/mL, respectively. Urinary BLCA-4 level was significantly higher in the NMIBC group than in the other 2 groups (*P* < 0.01); meanwhile, the BPH group was higher than the normal control group (*P* < 0.05). Median serum BLCA-4 levels in the NMIBC, BPH, and normal control groups were 5.680 ng/mL, 5.928 ng/mL, and 5.473 ng/mL, respectively, showing no significant difference among groups (*P* > 0.05).

**Conclusion:**

As a new marker of bladder cancer, urinary BLCA-4 level detection might apply for clinical diagnosis or postoperative monitoring for NMIBC.

## 1. Introduction

Bladder cancer is one of the most common urinary tumors, and the incidence is increasing with industrial development, increased smoking, and population aging [1]. Worldwide, bladder cancer has the sixth highest incidence rate among cancers in men [2]. Non-muscle-invasive bladder cancer (NMIBC) lacks specific symptoms and has a high recurrence rate [3]. Therefore, improving early diagnosis and long-term postoperative monitoring of bladder cancer has become a focus of domestic and international research.

With developments in molecular biology and immunology, numerous bladder cancer markers have emerged. Bladder cancer–specific nuclear matrix proteins (BLCAs) are specifically expressed in cancer tissue and can be released into the body during cell lyses [4]. Measurement of the BLCA level in body fluids with modern immunologic methods might be valuable to diagnose and monitor patients with bladder cancer. Current studies [5–7] state that BLCA-4 has relatively high sensitivity and specificity for bladder tumor diagnosis, presenting a high potential for clinical application. Konety et al. [8, 9] employed an indirect enzyme-linked immunosorbent assay (ELISA) to determine the urinary BLCA-4 level in patients with bladder cancer and found that with a cutoff value of 13 optical density units/*μ*g protein, sensitivity and specificity for tumor detection were 96.4% and 100%, respectively, and that urinary BLCA level was irrelevant for tumor staging and grading. In another study, Feng et al. [10–12] prepared a BLCA-4 antigen and used indirect ELISA to test urine samples of patients with bladder cancer and found that with a cutoff value of 1.7 × 10^−4^ optical density units/*μ*g protein, sensitivity and specificity for tumor detection were 97.37% and 100%, respectively. They also found that the urinary BLCA-4 level was associated with bladder tumor stage and that patients with advanced MIBC had a higher BLCA-4 level. All the above-mentioned studies demonstrated good sensitivity and specificity as well as high application potential of urinary BLCA-4. However, there are no standards for ELISA, the cutoff value is not unified, and the correlation between the urinary BLCA-4 level and tumor stage or grade requires further investigation.

The role of serum or urine BLCA-4 in NMIBC detection has not been described yet. Therefore, in this study, we assessed the levels of BLCA-4 in both serum and urine from NMIBC patients, benign prostate hyperplasia (BPH) patients, and healthy volunteers to evaluate its diagnostic value.

## 2. Materials and Methods

### 2.1. Subject Inclusion and Grouping

Among inpatients treated in our hospital's Department of Urology from December 2012 to May 2013, 60 patients were randomly selected. Twenty subjects with primary NMIBC were included in the experimental group (14 men, 6 women; mean age 59.10 ± 12.72 years, range 35–79 years). According to the 2004 World Health Organization grading method, 2 subjects had papillary urothelial neoplasm of low malignant potential, 13 had low-grade tumors, and 5 had high-grade tumors. According to the 2009 tumor node metastasis staging standard, 14 subjects were in stage Ta and 6 were in stage T1. Blood and urine samples were collected from all patients after admission, while cancer and adjacent tissue samples were collected during transurethral or open resection. Control group A was the normal control group (20 men with routine physical examination in the outpatient clinic, mean age 37.60 ± 13.51 years, range 21–67 years). Blood and urine samples were collected from all patients. Control group B comprised 20 patients with benign prostatic hyperplasia (BPH) (mean age, 67.75 ± 5.57 years, range 58–79 years). Blood and urine samples were collected preoperatively, while normal bladder wall tissue samples were collected intraoperatively. Patients were excluded if they met any of the following criteria: recurrent bladder cancer; cystoscopy, urinary catheterization, or urethral dilatation within 1 week; concurrent urinary stones, urinary tract infection, other urinary tract inflammatory stricture, or neoplastic disease; other severe disease and inability to tolerate surgery; or autoimmune disease, such as systemic lupus erythematosus or rheumatism arthritis. The study was approved by the Medical Research Ethics Committee, and all subjects provided written informed consent.

### 2.2. Sample Collection

On the morning after admission, 10 mL of midstream urine was collected, and a non-anticoagulant tube was used to collect 5 mL of cubital venous blood from each patient. Patients in the experimental group underwent transurethral resection of the bladder tumor or open resection at 2 cm from the tumor for complete resection, during which cancer and adjacent tissue samples were collected for pathologic and IHC testing. Patients in control group B underwent transurethral resection of the prostate or open resection. Full-thickness mucosal tissue samples from the posterior bladder wall, which were smooth and had no inflammatory congestion, ulcer, or other abnormity, were simultaneously collected for IHC testing.

### 2.3. Main Reagents and Instruments

Products were from the following vendors: human BLCA-4 kit (CSB-E14959h; Wuhan Cusabio Biotech, China); BLCA-4 antigen (Lot No. CPAH260174; Wuhan Cusabio Biotech, China); blocking solution, secondary antibody, horseradish peroxidase (HRP) streptavidin, and 3,3′-diaminobenzidine (DAB) color reagent (FXP020-060; Beijing 4A Biotech, China); tissue lysis solution (P0012E-1) and protease inhibitor (ST506; Beyotime Institute of Biotechnology, China); bicinchoninic acid (BCA) kit (serial No. PC0020) and ECL Plus Super-Enhanced chemiluminescence detection reagent (PE0010-100; Beijing Solarbio, China); Multiskan MK3 microplate reader, STP 120 tissue processor, and 171 tissue embedding station (Thermo Scientific); Leica RM2125 rotary manual microtome and Leica TK-218 thermostatic slice spreading and baking machine (Hubei Taiva Medical Technology, China); and Nikon Eclipse 80i biologic microscope (Shanghai Jiangwen Information Technology, China).

### 2.4. Determination of BLCA-4 Level in Urine and Serum Using Competitive ELISA

In a 96-microwell plate coated with anti-BLCA-4 antibodies, 50 *μ*L of sample (urine or serum) was added; standard solution was added as a positive control to generate a standard curve, and phosphate-buffered saline (PBS) was used as a negative control. After incubation at 37°C for 1 h, the plate was washed with PBS 3 times. Then, 50 *μ*L HRP-conjugated BLCA-4 was added and incubated at 37°C for 1 h. After 3-time washes, 50 *μ*L substrate A and 50 *μ*L substrate B were added and incubation was conducted at 37°C for 15 min. Finally, 50 *μ*L stop solution was added to each reaction well, and the plate was slightly knocked for sufficient mixing. Corresponding optical density values were read within 10 min using a microplate reader at a wavelength of 450 nm, and data were recorded. Average OD values were calculated, and BLCA-4 levels were determined using the standard curve for further analysis.

### 2.5. Determination of BLCA-4 Level in Cancer, Adjacent, and Normal Bladder Tissues Using Routine Pathology and IHC

Bladder tissue was embedded in paraffin, cut, deparaffinized, and hydrated, and the treated samples were placed in 3% H_2_O_2_ solution to incubate at 37°C for 30 min. Then, endogenous peroxidase of the tissue was inactivated, followed by washing with PBS for 5 min, 3 times. Goat serum working solution was added dropwise to the block at 37°C for 30 min. After the blocking solution was disposed, 1 : 100 diluted primary antibody was added dropwise, and the reaction could proceed overnight at 4°C. The next day, the insulated box was rewarmed at 37°C for 30 min, followed by washing with PBS for 5 min, 3 times. Biotinylated secondary antibody was added dropwise, and incubation was conducted at 37°C for 30 min, followed by washing thrice with PBS for 5 min. Then, HRP streptavidin was added dropwise, and incubation was conducted at 37°C for 30 min, followed by washing thrice with PBS for 5 min. After color development using DAB color reagent, a microscope was used for observation. A positive result was defined as BLCA-4 diffusely distributed in the nucleus with a yellowish-brown color. Image results were recorded for analysis.

### 2.6. Statistical Analysis

Intergroup comparisons of measurement data were conducted using the rank-transformed nonparametric test, analysis of variance, or independent-sample *t*-test. Intergroup comparisons of enumeration data, presented as odds ratios, were performed using Fisher's exact test. All data were processed using SPSS version 17.0 (SPSS Inc.), and *P* values of <0.05 were considered statistically significant.

## 3. Results

### 3.1. The Characteristics of the Participants

In the present study, a total of 60 participants, 20 patients with NMIBC, 20 with benign prostatic hyperplasia (BPH), and 20 normal controls, were included. Patients' characteristics are shown in Table 1.

### 3.2. BLCA-4 Differential Expression in the Bladder Cancer Tissues and the Control Benign Tissues

**Figure 1:**
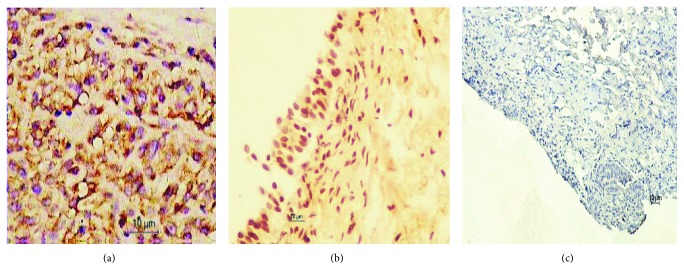
Immunohistochemistry images of BLCA-4 expression in the bladder cancer tissue (a), the adjacent normal tissue (b), and the normal tissue (c).

Expression of BLCA-4 in bladder cancer tissues, corresponding adjacent normal tissues, and normal bladder tissues from normal people was detected using the immunohistochemistry method. In general, the BLCA-4 staining intensity gradually increased from the normal tissue from normal people and benign adjacent tissue from the patient to the cancer tissue, as shown in Figure 1, and the BLCA-4 expression in cancer tissues was significantly higher than that in nontumor tissues of the other two groups (*P* < 0.001). These results suggested that upregulation of BLCA-4 was correlated with the benign bladder tissue cancerization.

### 3.3. BLCA-4 Level in Urine and Serum

**Figure 2:**
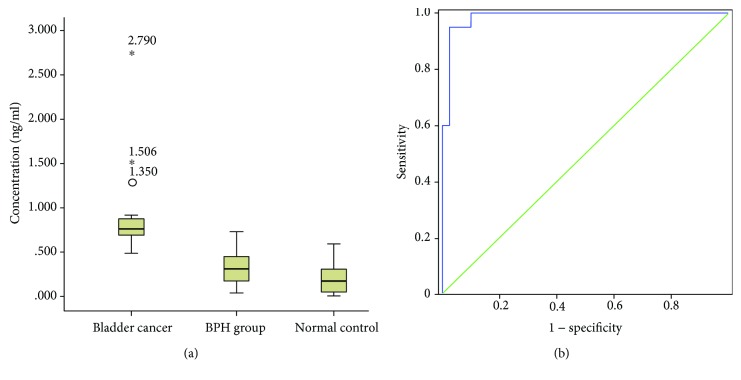
The diagnosis effect of urine BLCA-4 level on the bladder cancer. (a) BLCA-4 urine levels (ng/mL) of non-muscle-invasive bladder cancer (NMIBC) patients compared with benign prostatic hyperplasia (BPH) and normal individuals. (b) Receiver operating characteristic (ROC) curves for BLCA-4 distinguishing between NMIBC and non-NMIBC. The area under the curve (AUC) was 0.986 (95% confidence interval (CI) 0.963–1.000, *P* < 0.001). The cutoff value of 0.620 ng/mL was optimal for the detection of bladder cancer with a sensitivity of 95.0% and a specificity of 97.5%.

**Figure 3:**
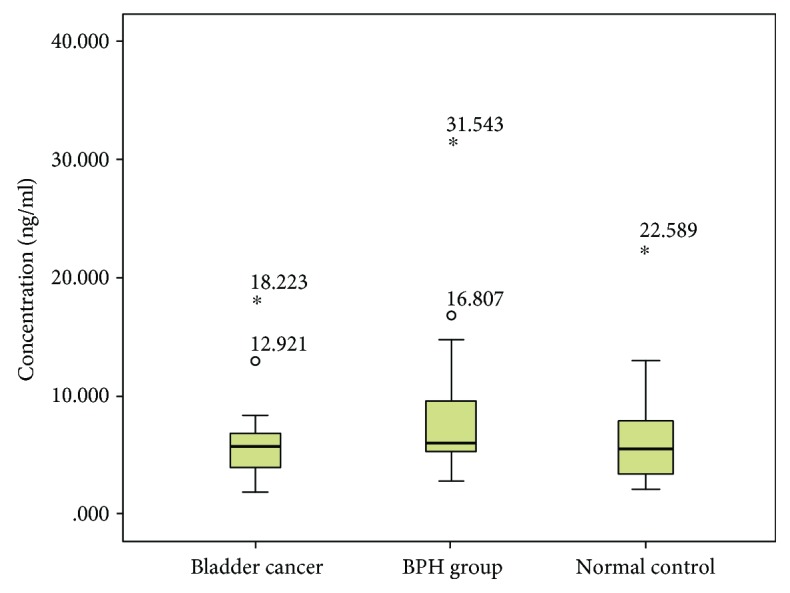
The BLCA-4 serum levels (ng/mL) of non-muscle-invasive bladder cancer (NMIBC) patients compared with benign prostatic hyperplasia (BPH) and normal individuals.

First, we detected if BLCA-4 levels differ in the urine of bladder cancer patients compared to BPH patients and healthy individuals. We found that the median urine BLCA-4 level was 0.759 ng/mL and significantly increased in the cancer patients compared with BPH patients (*P* < 0.01) and healthy people (*P* < 0.01) (Figure 2(a)). In addition, further analysis showed that the urinary BLCA-4 level in the cancer group had no significant difference based on sex, age, number of tumors, positive rate of adjacent tissue, stage, or grade (*P* > 0.05) (Table 2). Subsequently, we performed a receiver operating characteristic (ROC) analysis of the urine BLCA-4 level in order to test whether it could be a marker to distinguish between bladder malignancy and benign disorder and disease free. The area under the ROC curve (AUC) was 0.986 (98% CI, *P* < 0.001) (Figure 2(b)). The ROC curve that was plotted using the urinary BLCA-4 data of control group A as the control showed that with a cutoff value of 0.620 ng/mL, a sensitivity of 95.0% (19/20) and a specificity of 97.5% (39/40) for detection of bladder cancer were optimal (Figure 2(b), Table 3). Then, we examined whether the serum BLCA-4 had the same or similar role on the diagnosis of the bladder cancer. As shown in Figure 3, the serum BLCA-4 level was not significantly different among the 3 groups (*P* > 0.05). These data, therefore, indicated that the serum BLCA-4 was not suitable for the diagnosis of the bladder cancer, although its mechanism should be studied further.

## 4. Discussion

Currently, there are numerous tumor markers for bladder cancer; however, none have a satisfactory sensitivity or specificity. BLCA-4 is a nuclear matrix protein specifically expressed in bladder tumor tissue, discovered by Getzenberg et al. [4] in 1996 using proteomic technology. Ji et al. [13] studied the DNA segments of BLCA-4 in the blood leukocytes of patients with bladder cancer using a polymerase chain reaction method. They found that the population with BLCA-4 hypomethylation was at higher risk of bladder cancer and that the prognosis of patients with bladder cancer could be determined by evaluating the degree of DNA methylation. Using the IHC method, Feng et al. [10] found that BLCA-4 was located mainly in the nucleus of tumor cells and that there were no correlations between BLCA-4 and tumor grade or stage. Zhao et al. [14] found that BLCA-4 in tumor tissue existed primarily in the nucleolus and that its expression was not associated with sex, age, or tumor size. On the basis of these findings, we may expect that levels of BLCA-4 will be altered at the bladder carcinogenesis.

The cells in bladder cancer tissue grow and metabolize quickly, and the nuclear matrix protein can be released into the blood and urine during apoptosis, forming the molecular basis on which immunologic methods can be used to detect bladder cancer–specific nuclear matrix proteins. In this study, competitive ELISA was used to determine the urinary BLCA-4 level in patients with NMIBC, patients with BPH, and subjects with a healthy urinary tract. The results indicated that the urinary BLCA-4 level was significantly elevated in the NMIBC group compared with the other 2 groups. According to the ROC curve, when the cutoff value for the urinary BLCA-4 level was set at 0.620 ng/mL, the sensitivity and specificity for NMIBC diagnosis were 95% (19/20) and 97.5% (39/40), respectively. Besides, expression of urinary BLCA-4 in patients with NMIBC showed no correlation with age, sex, number of tumors, or tumor size, stage, or grade, which is consistent with the results of a study conducted by Getzenberg et al. [4], and this ensured that the BLCA-4 detection was less affected with higher specificity and more reliable detection results.

In the present study, the urinary BLCA-4 level was significantly elevated in the BPH group compared with the normal control group, which is inconsistent with the results of Konety et al. [8]. The reasons could not be explained at present. The urinary BLCA-4 level was higher in patients with BPH than in normal subjects, which might be related to the presence of bladder stones and/or infection, which can easily cause lower urinary tract obstruction. After occurrence of bladder urothelial lesions, self-repair and self-reproduction capabilities increase under regulation of DNA and protein molecules. Because BLCA-4 is most like ELK-3 of the ETS-domain transcription factor family and because it promotes cell apoptosis and proliferation, it can increase the BLCA-4 level in patients with BPH [15]. However, the level is still far lower than that in patients with bladder cancer; therefore, it would not affect the diagnosis of the cancer.

## 5. Conclusions

In summary, we show that BLCA-4 was significantly increased in the urine of the NMIBC and a little increased in BPH patients compared to those bladder- or prostate-disease-free individuals. The results of our study demonstrated that urine BLCA-4 might serve as a diagnostic marker to distinguish NMIBC. Finally, there was an obvious limitation in this study, which was the 20-sample size of each group. Future studies with a larger number of samples are needed to confirm these findings further.

## Figures and Tables

**Table 1:** Characteristics of the participants.

Characteristics	Groups (*N* = 20 each)		
	NMIBC	BPH	Normal
Age, y (mean, range)	59.1, 35–79	67.7, 58–79	37.6, 21–67
Gender (male, %)	14, 70%	11	20, 100%

NMIBC: non-muscle-invasive bladder cancer; BPH: benign prostatic hyperplasia.

**Table 2:** Comparison of BLCA-4 expression in non-muscle-invasive bladder cancer tissue with different clinical and pathologic features.

Parameter		No. of subjects	BLCA-4 expression, INT ∗ mm^2^	*P* value
Tumor size, cm	≥2	9	295.42 ± 15.37	0.436
	<2	11	305.46 ± 25.64	
Grade	Papilloma	2	297.53 ± 9.82	0.268
	Low	13	301.56 ± 18.62	
	High	5	314.27 ± 19.53	
Stage	Ta	14	304.26 ± 18.64	0.501
	T1	6	299.57 ± 16.25	

All values are reported as mean ± SD.

**Table 3:** Sensitivity and specificity of urinary BLCA-4 detection using a competitive enzyme-linked immunosorbent assay with different cutoff values.

Cutoff value (ng/mL)	Sensitivity	Specificity
0.483	1.000	0.900
0.589	0.950	0.925
0.620	0.950	0.975
0.637	0.900	0.975
0.730	0.700	1.000

## Data Availability

The original data used to support the findings of this study are available from the corresponding author upon request.
